# Homozygous and Heterozygous Nuclear Lamin A p.R582C Mutation: Different Lipodystrophic Phenotypes in the Same Kindred

**DOI:** 10.3389/fendo.2018.00458

**Published:** 2018-08-20

**Authors:** Renan Magalhães Montenegro, Aline Dantas Costa-Riquetto, Virgínia Oliveira Fernandes, Ana Paula Dias Rangel Montenegro, Lucas Santos de Santana, Alexander Augusto de Lima Jorge, Lia Beatriz de Azevedo Souza Karbage, Lindenberg Barbosa Aguiar, Francisco Herlânio Costa Carvalho, Milena Gurgel Teles, Catarina Brasil d'Alva

**Affiliations:** ^1^Brazilian Group for the Study of Inherited and Acquired Lipodystrophies, Faculdade de Medicina, Universidade Federal do Ceará, Fortaleza, Brazil; ^2^Monogenic Diabetes Group, Genetic Endocrinology Unit (LIM25), Hospital das Clinicas da Faculdade de Medicina, Universidade de São Paulo, São Paulo, Brazil

**Keywords:** Dunnigan, partial lipodystrophy, generalized lipodystrophy, LMNA, FPLD2, phenotypic heterogen

## Abstract

**Background:** Dunnigan-type familial partial lipodystrophy (FPLD2) is a rare autosomal dominant disease caused by heterozygous mutations in the *LMNA* gene that results in regional loss of subcutaneous adipose tissue with onset in puberty. However, a generalized lipodystrophy phenotype has also been associated with heterozygous mutations in this gene, demonstrating the noticeable phenotypic heterogeneity of this disease.

**Methods:** We report and describe clinical and metabolic features of four patients from the same family with the p.R582C *LMNA* mutation, three homozygous and one in the heterozygous state that present with three distinct lipodystrophic phenotypes.

**Results:** Case description: The proband was a 12-year-old girl who developed severe subcutaneous fat atrophy in limbs and abdomen followed by a remarkable dorsocervical fat accumulation in adulthood along with diabetes at age 23. The proband's sister was a phenotypically normal girl who developed hypertriglyceridemia at age 8, progressive features of partial lipodystrophy at age 11, and diabetes at age 22. The proband's mother was first examined at age 32, presenting diabetes and a severe generalized lipodystrophic phenotype; she developed kidney failure at age 41 and died due to diabetic complications. The proband's father was a 50-year-old man with abdominal fat concentration that was initially considered phenotypically normal. Massively parallel sequencing using a platform of genes related to genetic lipodystrophies, followed by Sanger sequencing, revealed the transversion c.1744C>T at exon 11 of the *LMNA* gene (p.R582C) in the homozygous (mother and daughters) and heterozygous (father) states.

**Conclusion:** We documented three distinct phenotypes of the homozygous and heterozygous p. R582C *LMNA* mutation in the same kindred, illustrating that FPLD2 linked to mutations in this gene is a disease of great clinical heterogeneity, possibly due to associated environmental or genetic factors.

## Introduction

Dunnigan-type familial partial lipodystrophy (FPLD2; OMIN 151660) is a rare autosomal dominant disease characterized by regional loss of subcutaneous adipose tissue (limbs and trunk) that occurs progressively during peripubertal phase and is often associated with conspicuous insulin-resistance, diabetes, and other metabolic disturbances later in life (1, 2). Normal stores of fat are found in the bone marrow as well as intra-abdominal and intra-thoracic regions and excess fat may be deposited in the face, neck, back, and labia majora (2).

FPLD2 is attributed to heterozygous missense mutations in the *LMNA* gene (MIM150330) on chromosome 1q21–22 (3–5). Through alternative splicing, *LMNA* encodes lamins A and C, which are intermediate filament proteins consisting of a short N-terminal head, a central α-helical rod domain, and a large C-terminal tail containing a globular immunoglobulin-like domain; they form polymers at the nuclear lamina—a structural meshwork located in the inner aspect of the nuclear envelope—and interact with chromatin and integral proteins through binding sites in the rod domain and C-terminal tail. Lamins are regulators of structure, shape, and mechanical stability of the nucleus and have roles in DNA replication and repair, chromatin organization, spatial rearrangements of nuclear pore complexes, nuclear growth, and anchorage of nuclear envelope proteins (3).

Naturally occurring mutations in the *LMNA* gene have been described in distinct disease phenotypes called laminopathies whose spectrum includes FPLD2, skeletal and cardiac myopathies, neuropathies, premature aging syndromes, and overlapping syndromes (6–11). In FPLD2, the heterozygous amino acid changes are mostly located in the C-terminal domain of lamin A/C with a mutational hot-spot that substitutes the positively charged arginine at codon 482 with a neutral amino acid (4).

Two mechanisms were proposed to explain how *LMNA* mutations result in the observed diseases. *LMNA* mutations either impair the ability of the lamina in transmitting external mechanical signals into the nucleus and maintaining nuclear integrity (mechanical model) or impair the capacity of lamins in interacting with transcriptional regulators (gene expression model) (12). FPLD2-causing mutations possibly disrupt gene regulation, although the precise mechanism for this dysregulation and the resulting tissue manifestations are unclear. Recently, after discovering that miR-335 inhibits proliferation and differentiation of human mesenchymal stem cells into osteogenic and adipogenic lineages, it has been shown that this miRNA also plays a role in the etiology of FPLD2 (13). The heterozygous R482W mutation of the *LMNA* gene impairs the ability of lamin A in silencing the MEST/miR-335 locus in human adipose stem cells, resulting in elevated levels of the anti-adipogenic factor miR-335 observed in FPLD2 (14, 15).

In this report, we describe two severe and distinct lipodystrophic phenotypes associated with the p.R582C *LMNA* mutation in the homozygous state (one of them with generalized lipodystrophic features) and a less severe phenotype in a heterozygous carrier in the same family.

## Materials and methods

Clinical features as well as the laboratory and molecular data of four related patients are presented herein. This study was approved by the ethics committee of the Universidade Federal do Ceará, Fortaleza, Brazil. The patients gave written informed consent for the study and for publication of their clinical, biochemical, and molecular data.

Blood samples were obtained after overnight fast. Blood glucose, cholesterol, and triglyceride levels were determined according to standard methods using automated equipment, serum insulin levels were determined by immunoassays with reagents provided by Roche Diagnostics (Basel, Switzerland), and hemoglobin A1c values were measured by ion exchange high performance liquid chromatography (HPLC). Plasma leptin levels were determined by an immunoassay using a commercial kit (DIAsource Immunoassays, Louvain-la-Neuve, Belgium).

Body fat distribution was assessed using whole body magnetic resonance imaging (MRI) performed in a 1.5-Tesla imaging device (GE Signa HDxt; Waukesha, Wiscosin). Pre-selected body segments were surveyed using contiguous axial and coronal 3.5-mm slices, a T1-weighted spin-echo sequence, and in-phase and out-of-phase T1 sequences for abdominal studies.

DNA was extracted from peripheral blood leucocytes using standard in-house protocols based on a salting-out method (16). The proband's mother was analyzed by a customized massively parallel sequencing panel to capture the coding regions and intron-exon boundaries of the following genes related to genetic lipodystrophies, *AGPAT2, BSCL2, CAV1, PTRF, LMNA, PLIN1, ZMPSTE24, AKT2, CIDEC, TBC1D4*, and *PPARG*. The panel included 51 genes related to monogenic forms of diabetes in addition to the mitochondrial genome (17). Length of the target region was approximately 488 kb, which spans all exons examined. Paired-end sequencing (2 × 150 bp) was performed using an Ilumina MiSeq platform (Illumina, San Diego, CA). Bioinformatics analysis was executed at the Laboratório de Sequenciamento em Larga Escala da FMUSP (SELA), Sao Paulo, Brazil using in-house analysis pipelines. Pathogenicity of identified variants was classified according to the American College of Medical Genetics and Genomics and the Association for Molecular Pathology (ACMG/AMP) guidelines (18). After confirming the genetic diagnosis of the mother, the husband and daughters were investigated using Sanger sequencing (19).

## Results

### Study subjects

#### Patient 1 (proband)

**Figure 1:**
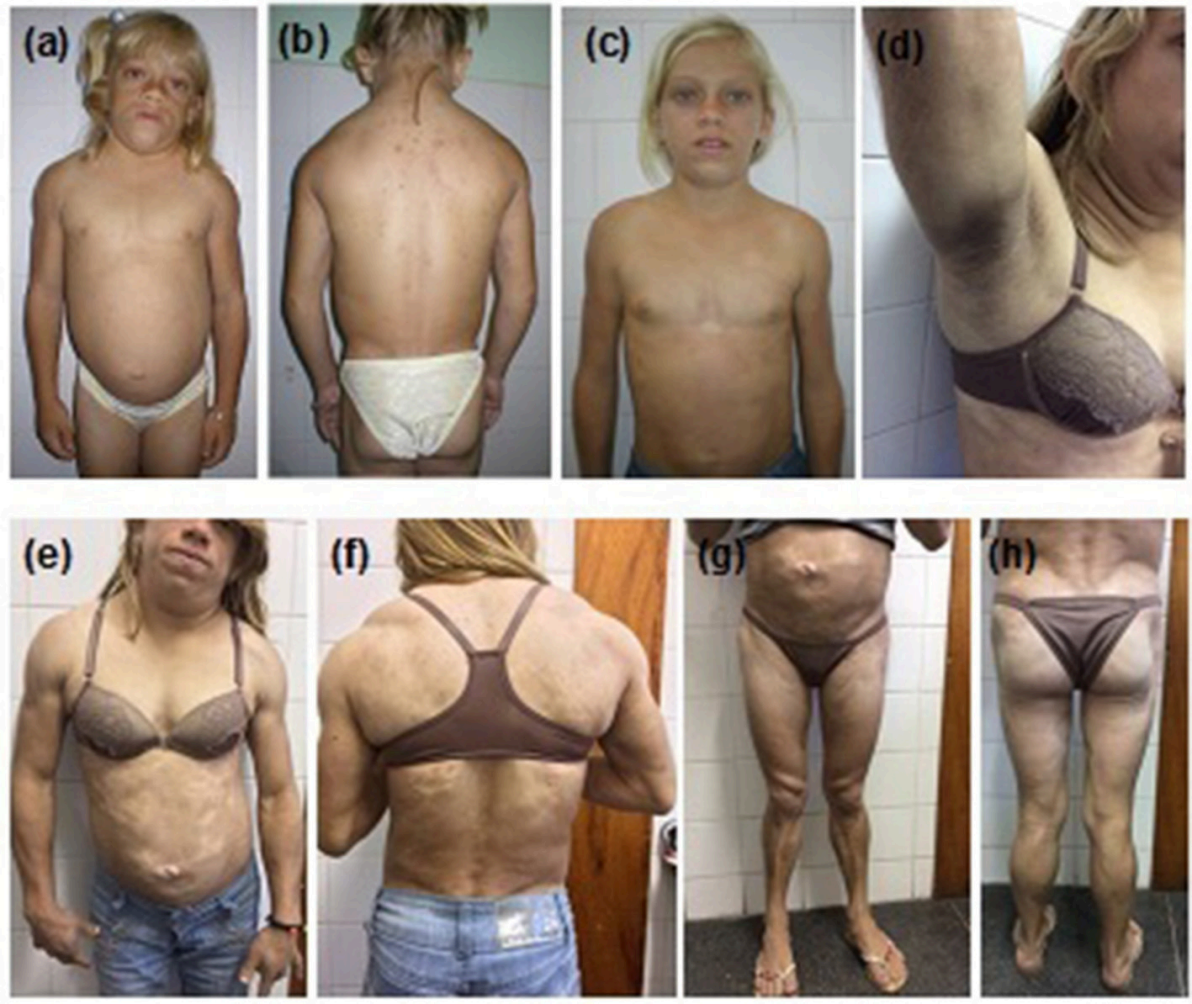
Patient 1 (proband) with familial partial lipodystrophy bearing the homozygous p.R582C lamin A mutation at **(a,b)** age 12 with decompensated hypothyroidism. **(c)** One year after levothyroxine treatment. Adulthood **(d)** acanthosis nigricans, **(e,f)** fat accumulation in the neck and dorsocervical region, and **(g,h)** loss of subcutaneous tissue in the limbs and abdomen. Morphea hypochromic cutaneous lesions can be seen in the abdomen and trunk.

The proband (Figure 1) was a 12-year-old girl, born at full-term (birth weight, 3,600 g) as the first child of consanguineous parents, referred to the Universidade Federal do Ceará Clinical Hospital, Fortaleza, Brazil for clinical assessment of short stature and learning disabilities that manifested since age 8. Physical examination revealed reduced fat in the arms, legs, and gluteal region, muscular hypertrophy, and acanthosis nigricans as well as macroglossia, dry and thickened skin, short stature, and pubertal stage Tanner 1. Proband was 118.7 cm (Z-score, −5.2) in height, weighed 27 kg, and had a body mass index (BMI) of 19.1 kg/m^2^. Thyroid function tests revealed severe primary hypothyroidism (TSH > 100 uU/mL and free T4 = 0.01 ng/dL) and thus the proband was started on levothyroxine resulting in catch-up growth and normal pubertal development with menarche by age 14.8. After hypothyroidism treatment, the selective loss of subcutaneous fat tissue in limbs, gluteal region, and abdomen became evident over time, leading to the diagnosis of lipodystrophy. At age 12, she presented with moderate hepatomegaly, umbilical hernia, hypertriglyceridemia (509 mg/dL), and acanthosis nigricans in the neck and axillary regions. One year later, at age 13, hypochromic and atrophic cutaneous plaques were observed distributed throughout the body. Skin biopsies revealed localized scleroderma (morphea). Some years later in adulthood, remarkable fat accumulation in the neck, face, and axillary and dorsocervical regions was observed, along with the worsening of subcutaneous fat atrophy in limbs and abdomen, indicating partial lipodystrophy diagnosis.

At age 23, the proband was diagnosed with diabetes and albuminuria. Currently, she is 26 years old and presents with uncontrolled diabetes, hepatomegaly (10 cm below the costal margin), and irregular menses. She is 145 cm in height, weighs 42 kg, and has a BMI of 20.0 kg/m^2^. A formal assessment of intelligence quotient is not available, but she shows a slight degree of intellectual impairment. Standard serum determinations are listed in Table 1.

**Table 1:** Biochemical tests of patients with familial lipodystrophy.

**Patient**	***LMNA* Genotype**	**Age**	**BMI kg/m^2^**	Leptin ng/mL^*^	**TC mg/dL**	**HDL mg/dL**	**LDL mg/dL**	**TG mg/dL**	**Glycemia mg/dL**	**A1c %**	**AST U/L**	**ALT U/L**	**GGT U/L**	**Albumin/creatinine mg/g**
1. Proband	Homozygous	26	20.5	1.8	236	30	136	334	161	8.8	145	118	–	74
2. Sister	Homozygous	22	20.7	1.8	177	33	113	156	149	7.0	109	135	–	11.7
3. Mother	Homozygous	32	17.2	1.2	188	38	–	796	390	15.5	49	70	47	–
4. Father	Heterozygous	50	24.9	1.9	216	37	146	164	70	4.8	20	20	52	20.3

* Reference values of leptin (BMI 18–24 kg/m^2^): 0.5–3.2 ng/mL; (BMI 25–29 kg/m^2^): 0.5–14.6 ng/mL.

*BMI, body mass index; TC, total cholesterol; HDL, high-density lipoprotein; LDL, low-density lipoprotein; AST, aspartate aminotransferase; ALT, alanine aminotransferase; GGT, gamma-glutamyl transferase*.

#### Patient 2 (proband's sister)

**Figure 2:**
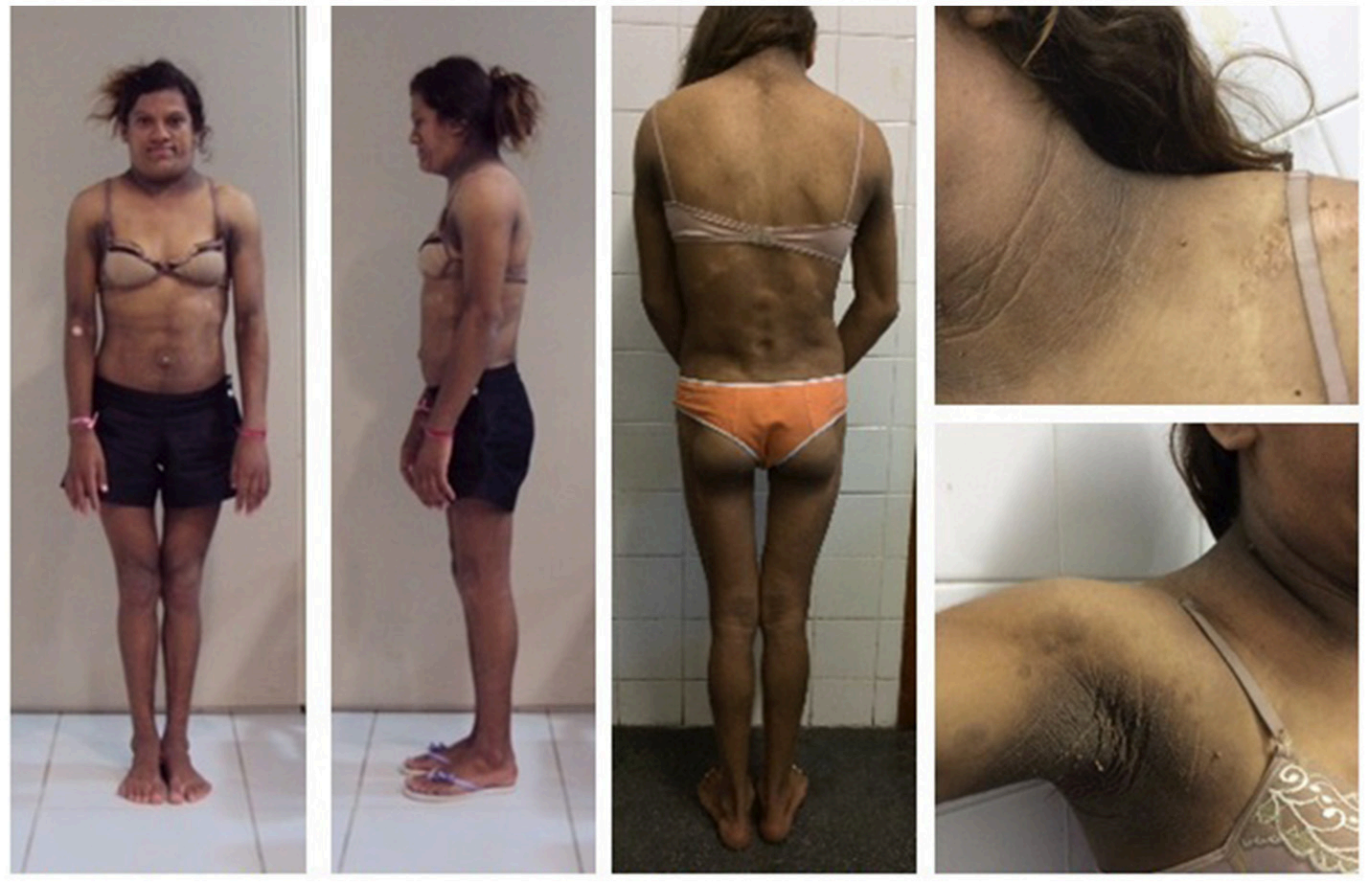
Patient 2 (proband's sister) with familial partial lipodystrophy bearing the homozygous p.R582C lamin A mutation in adulthood showing loss of subcutaneous tissue in limbs and abdomen, fat accumulation in neck and dorsocervical region, and notable acanthosis nigricans.

The proband's sister (Figure 2) was born at full-term with a birth weight of 4,800 g. She regularly accompanied her sister to the medical visits and was a phenotypically normal 8-year-old girl with hypertriglyceridemia (316 mg/dL) when the proband was first examined. By age 11, the proband's sister had started thelarche and after the onset of puberty, subtle clinical features of partial lipodystrophy became apparent. Her age at menarche was 13. Subcutaneous fat progressively disappeared from the limbs, gluteal region, and abdomen while fat accumulation became evident in the face, neck, and axillary and dorsocervical regions. She also developed intense acanthosis nigricans in the neck and axillary regions. At age 15, atrophic and hypochromic cutaneous plaques were found diffusely distributed and a biopsy was consistent with localized scleroderma.

Currently, she is 22 years old and has irregular menses, hepatomegaly (6 cm below the costal margin), and was recently diagnosed with diabetes. She is 163 cm in height, weighs 55 kg, and has a BMI of 20.7 kg/m^2^. Standard serum determinations are listed in Table 1.

#### Patient 3 (proband's mother)

**Figure 3:**
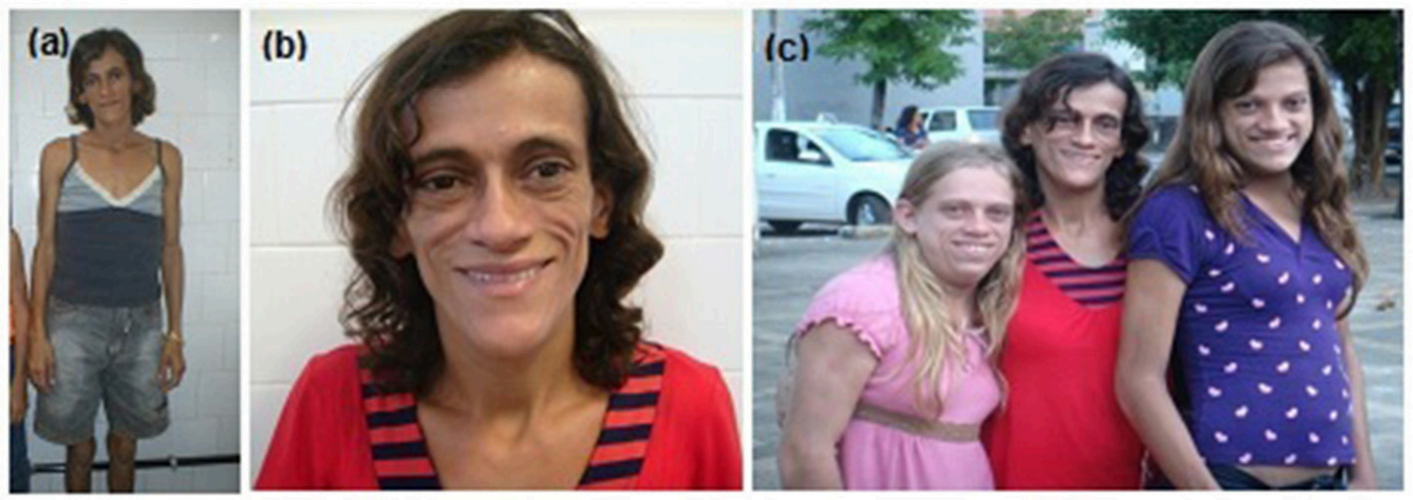
Patient 3 (proband's mother) bearing the homozygous p.R582C lamin A mutation **(a,b)** at age 32 showing a phenotype of apparently generalized lipodystrophy without fat accumulation in the neck and face and without acanthosis nigricans. **(c)** Mother and daughters (from L–R: patients 1, 3, and 2) at ages 19, 32, and 15 years old (L–R).

When the proband was first examined at our unit, a clinical examination of her mother (Figure 3) at age 32 revealed a severe generalized lipodystrophy phenotype. She relayed that this phenotype was present from birth and that her birth weight was 2,600 g. She underwent menarche at age 14, had regular menstrual periods, and was diagnosed with diabetes at age 24. She was 152 cm in height, weighed 40.4 kg, and had a BMI of 17.5 kg/m^2^. She had no signs of acanthosis nigricans and her liver was palpable 3 cm below the costal margin. Serum determinations at first visit are listed in Table 1. Subsequently during follow-ups, she underwent many hospitalizations due to uncontrolled diabetes, gluteal abscess, a foot ulcer, and toe amputation. At age 41, she was diagnosed with kidney failure and 1 year after receiving hemodialysis treatment, she died at age 42 due to diabetic complications.

#### Patient 4 (proband's father)

**Figure 4:**
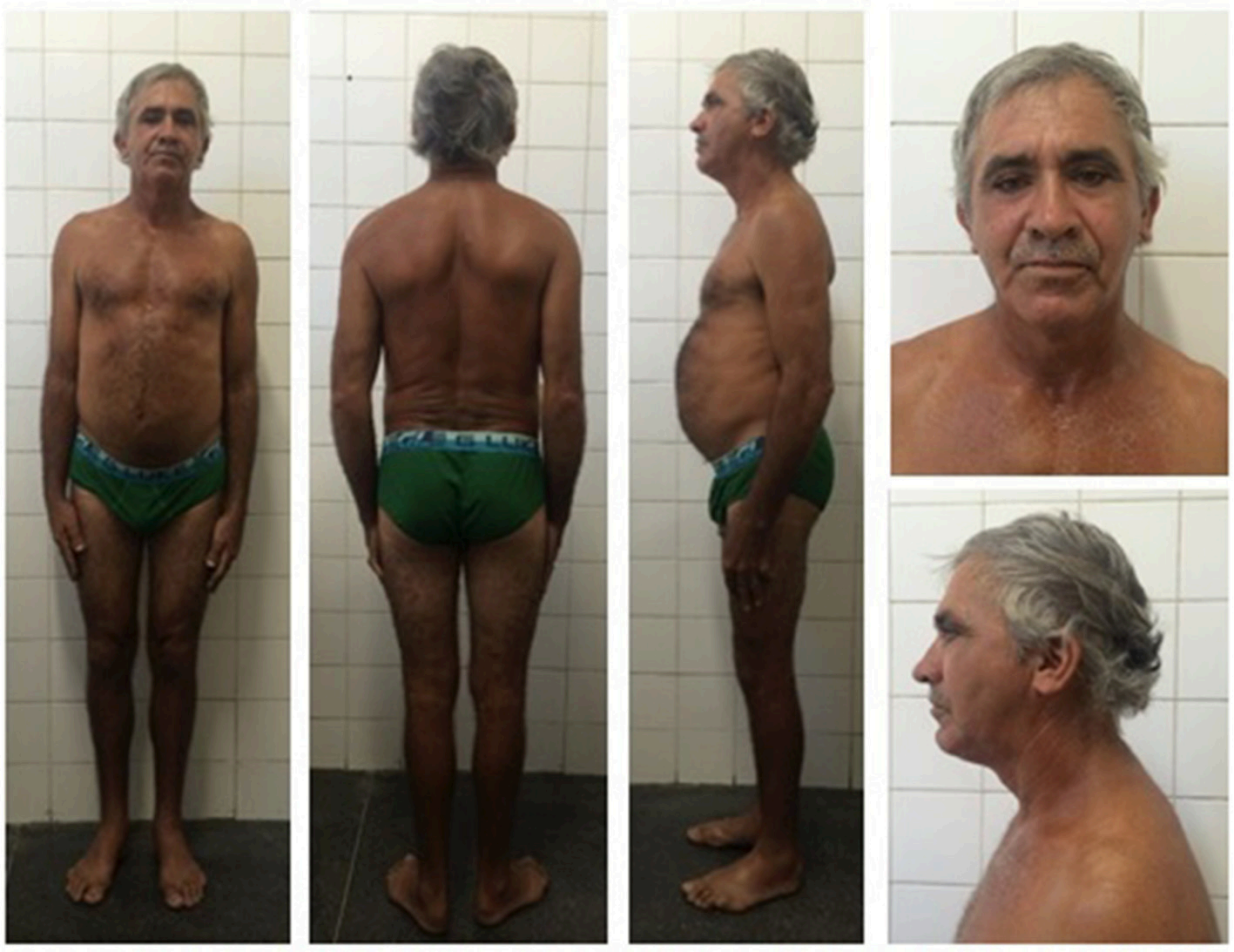
Proband's father bearing the heterozygous p.R582C lamin A mutation at age 50 and showing a subtle decrease in subcutaneous fat in the limbs and fat accumulation in the abdomen, without fat accumulation in the neck.

**Figure 5:**
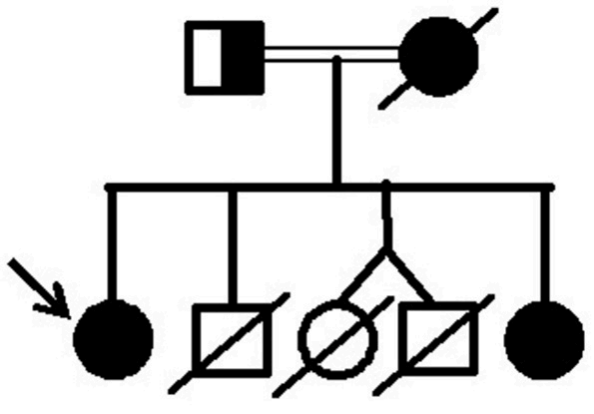
Pedigree of the family with lipodystrophy due to the p.R582C lamin A mutation.

Initial clinical examination of the proband's father showed no evidence of lipoatrophy. He is a 50-year-old man with abdominal fat concentration, weighs 72.2 kg, is 170 cm in height, and has a BMI of 24.9 kg/m^2^ (Figure 4). Standard serum determinations are listed in Table 1 and the pedigree of the family is shown in Figure 5.

### Molecular results

Massively parallel sequencing using the platform of genes related to genetic lipodystrophies revealed that the proband's mother had a homozygous genotype for the transversion c.1744C>T located at exon 11 of the *LMNA* gene, specifically encoding the lamin A isoform. This mutation leads to the substitution of arginine at position 582 by a cysteine (p.R582C).

Molecular studies of the daughters' genomes were performed using Sanger sequencing, which confirmed the p.R582C variant in the homozygous state. The proband's father was also tested and was found to have the same p.R582C but in a heterozygous state.

The c.1744C>T/p.R582C variant has been described as pathogenic by Mory et al. (20) and is registered in the Human Gene Mutation Database (HGMD) under the code CM124985.

### MRI results

**Figure 6:**
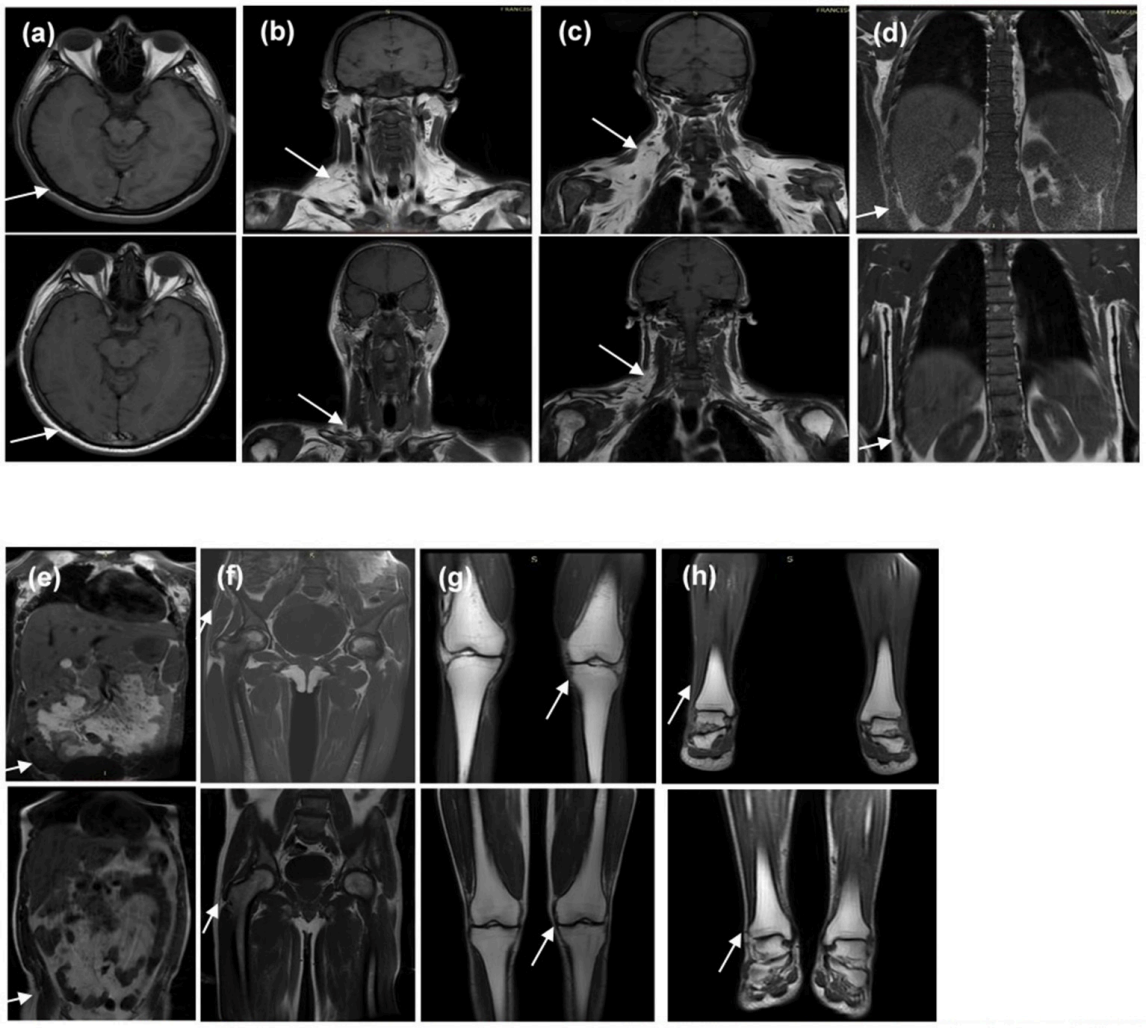
T1-weighted magnetic resonance imaging of the (1) homozygous proband's sister and (2) the heterozygous father at the levels of **(a)** orbits and scalp, **(b)** anterior neck, **(c)** dorsocervical, **(d)** thoracolumbar spine, **(e)** abdomen, **(f)** pelvis and thighs, **(g)** knees, and **(h)** feet. It is worth noting that the sister and father's body mass indices are 20.7 and 24.9 kg/m^2^, respectively. Note the diffuse absence in subcutaneous tissue of the homozygous proband's sister (indicated by the arrows in **a,d–h**) and excess fat deposition in the neck and dorsocervical area (indicated by the arrows in **b,c**).

An MRI scan was available for patient 2 (proband's sister). It showed near total absence of subcutaneous fat throughout the body in the analyzed body segments and preserved fat in the palms, soles, labia majora, and scalp. Retro-orbital fat was also present. Intraperitoneal, retroperitoneal, and perirenal fat was well preserved and there was a marked accumulation of fat in deep cervical regions as well as in the infraclavicular, axillary, and scapular regions. Bone marrow fat was markedly reduced in the spine and humerus, relatively preserved in the proximal femur, and normally preserved in the distal femur, tibia, fibula, and feet (Figure 6).

The MRI scan of patient 4 (proband's father) showed a less severe loss of subcutaneous fat throughout the body and preserved fat in the palms, soles, scalp, and retro-orbital regions. Intraperitoneal, retroperitoneal, and perirenal fat was well preserved and there was a less severe fat accumulation in the deep cervical regions in comparison with his daughter. Bone marrow fat was also markedly reduced in the spine and humerus (Figure 6).

## Discussion

Familial partial lipodystrophy of the Dunnigan type is an autosomal dominant disease with noticeable phenotypic heterogeneity.

All four family members were found to harbor the p.R582C mutation in nuclear lamin A. This variant has been previously reported in the heterozygous state in a 57-year-old Brazilian woman with decreased subcutaneous leg fat and a remarkable increase in trunk fat, a BMI of 30 kg/m^2^, 34.6% total body fat, 32.9 μg/mL leptin, who was diagnosed with dyslipidemia and hypertension at age 36 and diabetes at age 43 (20). This patient came from a distinct region of Brazil and is apparently unrelated to the patients in this study, although a founder effect was not studied in these families.

We identified three distinct phenotypic variants in our patients. Patient 1 and 2 (daughters) show typical partial lipodystrophy while patient 3 (proband's mother) showed generalized lipoatrophy. The generalized and partial lipodystrophic phenotypes associated with the same mutation in a homozygous state is remarkable, suggesting that other genetic factors are modulating clinical expressivity of this disease. On the other hand, it is also possible that the apparent generalized phenotype is a result of poor nutritional status as the patients come from a poor region and have a low family income. The low BMI (17.2 kg/m^2^) of patient 3 (proband's mother) may have prevented the typical fat accumulation of FPLD2, thereby masking the disease, highlighting the diagnostic difficulties regarding nutritional status especially in underdeveloped regions where undernutrition is a problem.

While the three women were homozygous for the p.R582C mutation, patient 4 (proband's father) was a heterozygous carrier initially considered phenotypically normal. After discovering that the father harbors the same mutation in a heterozygous state, a more careful clinical evaluation revealed a less severe phenotype of partial lipodystrophy that was confirmed by MRI. This phenotype is comparable to the patient described by Mory et al. (20), a carrier of the same heterozygous mutation and designated by the authors as “atypical partial lipodystrophy,” which is a less severe loss of adipose tissue with less severe metabolic abnormalities. It is possible that this common phenotype characterized by thin legs and arms, scarcity of fat in the gluteal regions, and excess fat in the abdomen and trunk, often associated with metabolic disturbances and known as a Cushingoid pattern, may be part of the spectrum of body fat distribution in the general population. The phenotype of body fat distribution possibly results from a myriad of genetic variation—mainly single nucleotide polymorphisms (SNPs)—but may be related to more severe genetic variants linked to the pathological state of lipodystrophies in some cases. On the other hand, it is known that lipoatrophy diagnosis may be delayed in men owing to the less evident loss of subcutaneous fat. In fact, men are usually diagnosed after their female counterparts (21).

Whereas FPLD2 is an autosomal dominant disease, most mutations of the *LMNA* gene associated with a lipodystrophic phenotype were found in the heterozygous state. However, a few patients were described with homozygous mutations. Le Dour et al. (22) found the homozygous *LMNA* T655fsX49 mutation in seven probands from Reunion Island presenting with severe lipodystrophic syndrome, similar to that observed in typical FPLD2 due to the common heterozygous p.R482 substitutions. Eight relatives were heterozygous for the T655fsX49 mutation and two of them were clinically lipodystrophic with metabolic disturbances while the other six were completely asymptomatic, indicating that T655fsX49 *LMNA*-linked lipodystrophic syndrome has a codominant transmission with incomplete penetrance in heterozygotes. Andre et al. (23) compared 12 homozygous and 25 heterozygous individuals from Reunion Island harboring the T655fsX49 mutation and found that the lipodystrophic traits were more pronounced in the homozygous group as indicated by a BMI of 19.8 vs. 25.1 kg/m^2^, 16.1 vs. 31.2% total body fat, and 2.4 vs. 11.2 ng/mL serum leptin levels in homozygous and heterozygous patients, respectively. There was no mention of any patient with a generalized phenotype in their study. The frequency of diabetes, hypertension, dyslipidemia, and hepatic steatosis tended to be higher in the homozygous group but did not reach statistical significance. These findings confirm a dose-dependent effect of the *LMNA* p.T655fs^*^49 mutation in affected patients.

Other cases of homozygous mutations of the *LMNA* gene related to lipodystrophic phenotypes have been published. Mory et al. (20) described a 26-year-old woman harboring the homozygous p.R584H *LMNA* substitution with a moderate reduction of subcutaneous fat in limbs and trunk, without submandibular or cervical fat accumulation, a BMI of 19 kg/m^2^, and low serum leptin (1.1 μg/mL) and HDL-cholesterol levels. This patient was classified by the authors as having typical FPLD2 and her parents were first-degree cousins heterozygous for this mutation. The father could not be evaluated, but he apparently did not exhibit the phenotypic alterations of lipodystrophy and had mild dyslipidemia (HDL-cholesterol, 35 mg/dL; LDL-cholesterol, 142 mg/dL). The mother had vitiligo lesions and dyslipidemia (HDL-cholesterol, 58 mg/dL; LDL-cholesterol, 189 mg/dL) but no evidence of lipoatrophy or acanthosis. The same p.R584H *LMNA* mutation has been previously described in the heterozygous state in two patients with familial partial lipodystrophy and metabolic complications such as diabetes, dyslipidemia, and hepatic steatosis (24, 25).

Regarding the apparent generalized lipodystrophy in patient 3 (proband's mother), heterozygous mutations in the *LMNA* gene have also been detected in patients with a more extensive form of pubertal-onset generalized lipodystrophy and severe metabolic abnormalities (26, 27). Mory et al. (26) described a 15-year-old girl with pubertal-onset generalized atrophy of subcutaneous fat, including palmar and plantar regions, BMI of 15 kg/m^2^, 8.6% body fat by dual-energy X-ray absorptiometry (DEXA), and severe metabolic disturbances; molecular studies revealed a heterozygous p.T10I mutation in the *LMNA* gene. Similarly, a 27-year-old man was described by Caux et al. (27) with generalized lipoatrophy of pubertal onset, BMI of 19.6 kg/m^2^, 8.6% body fat by DEXA, and severe metabolic disturbances caused by the heterozygous p.R133L *LMNA* mutation. Recently, Hussain et al. (28) described nine unrelated patients with generalized lipodystrophy due to the heterozygous T10I mutation; these mutations were present in the *LMNA* region encoding the N-terminal domain, while most of the mutations related to familial partial lipodystrophy are clustered in the C-terminal tail of this gene.

It is worth noting that the proband's mother (patient 3) and daughters (patients 1 and 2) were homozygous for this genetic variant. The parents of patient 3 were unrelated, but they are from a small country town in the state of Ceará in northeast Brazil that has 26,000 inhabitants and where consanguineous unions are frequent. On the other hand, patient 3 had a consanguineous marriage with a second-degree cousin, explaining the two homozygous daughters (patients 1 and 2).

The cutaneous manifestations of patients 1 and 2 are atypical. Interestingly, scleroderma skin lesions are present in some progeroid syndromes, such as Werner syndrome (WS) and Hutchinson-Gilford progeria syndrome (HGPS). HGPS has been attributed to *LMNA* gene mutations while WS is most frequently caused by mutations in the *RECQL2* gene, which is involved in DNA repair and telomerase maintenance process. However, some atypical cases of WS have been associated with heterozygous *LMNA* point mutations (29). This association suggests that the pathophysiological mechanism of scleroderma skin lesions may be related to abnormalities in the *LMNA* gene.

In this study, we identified a p.R582C mutation located in exon 11 of the *LMNA* gene affecting the C-terminal domain and specific for the lamin A isoform. Laminopathy phenotypes may be linked to the position of the *LMNA* mutation in relation to the nuclear localization sequence (NLS), which spans residues 416–423. Mutations associated with familial partial lipodystrophy are mostly downstream this point and in the C-terminal domain of lamin A/C (30); this domain of lamin A/C adopts an immunoglobulin fold-like structure and its tridimensional conformation is not disrupted by partial lipodystrophy associated mutations. Instead, these mutations alter the positive charge of the affected region, perturbing the interaction of lamin A/C with biological partners, such as transcriptional factors and regulators, and thus altering gene expression (31, 32).

In conclusion, we documented three distinct phenotypes of the p.R582C *LMNA* mutation in homozygous and heterozygous states in the same kindred. As this variant affects only lamin A and spares the other splice form, it may render a more severe phenotype when occurring in the homozygous state, suggesting a dose-dependent effect of the p.R582C *LMNA* mutation in affected patients. However, this mutation in a heterozygous state resulted in an uncertain phenotype and its homozygous state caused severe lipodystrophic syndromes with phenotypic differences. Our findings, as well as the previously described cases of generalized lipodystrophies due to *LMNA* mutations, illustrate that familial lipodystrophy linked to mutations in the *LMNA* gene is a disease of great clinical heterogeneity that is possibly due to associated environmental or genetic factors.

## Ethics statement

The protocol was approved by Walter Cantídio University Hospital Research Ethics Board, from Universidade Federal do Ceará, Fortaleza - Brazil. This study was carried out in accordance with the principles of the Declaration of Helsinki and written informed consent was obtained from all subjects.

## Author contribution

RM and Cd contributed to the conception of the work, acquisition, and interpretation of data, as well as writing and the manuscript review. AJ and MT contributed to the analysis and interpretation of data and reviewed the manuscript. VF, AM, and LK contributed to the acquisition of data and manuscript edit and review. LA, FC, AC-R, and LS contributed to the acquisition, analysis, and interpretation of data.

### Conflict of interest statement

The authors declare that the research was conducted in the absence of any commercial or financial relationships that could be construed as a potential conflict of interest.
